# A novel peptide mimetic, brilacidin, for combating multidrug-resistant *Neisseria gonorrhoeae*

**DOI:** 10.1371/journal.pone.0325722

**Published:** 2025-06-05

**Authors:** Abdallah S. Abdelsattar, Nader S. Abutaleb, Mohamed N. Seleem

**Affiliations:** 1 Department of Biomedical Sciences and Pathobiology, Virginia-Maryland College of Veterinary Medicine, Virginia Polytechnic Institute and State University, Blacksburg, Virginia, United States of America; 2 Center for One Health Research, Virginia Polytechnic Institute and State University, Blacksburg, Virginia, United States of America; University of Birmingham School of Dentistry, UNITED KINGDOM OF GREAT BRITAIN AND NORTHERN IRELAND

## Abstract

*Neisseria gonorrhoeae* is classified by the Centers for Disease Control and Prevention as an urgent public health threat due to rising infections and rapid resistance development. *N. gonorrhoeae* has developed resistance to nearly all FDA-approved drugs, with ceftriaxone being the only remaining effective treatment for gonococcal infections. Alarmingly, ceftriaxone-resistant *N. gonorrhoeae* strains were isolated worldwide, raising the potential of untreatable gonorrhea in the near future. Hence, the critical need to develop new anti-*N. gonorrhoeae* therapeutics cannot be overemphasized. In this study, we identified the peptide mimetic brilacidin as an effective anti-gonococcal agent. Brilacidin completed phase 2 clinical trials for treating skin infections, oral mucositis, and COVID-19. Herein, brilacidin displayed potent activity against a panel of 22 drug-resistant strains of *N. gonorrhoeae,* inhibiting 50% of the strains tested (MIC_50_) at the concentration of 4 µg/mL. The peptide exhibited rapid bactericidal activity, reducing *N. gonorrhoeae* high inoculum within two hours. Moreover, brilacidin was superior to the drug of choice, ceftriaxone, in eliminating the intracellular *N. gonorrhoeae* harbored within endocervical cells. Additionally, brilacidin showed high tolerability in mammalian cells and lacked hemolytic activity in human erythrocytes. Altogether, the results demonstrate that brilacidin is a promising anti-gonococcal agent that warrants further in-depth investigation.

## 1. Introduction

*Neisseria gonorrhoeae* is the bacterium responsible for gonorrhea, one of the most prevalent sexually transmitted diseases [1]. In the United States, the Centers for Disease Control and Prevention (CDC) estimates that 1.6 million new gonococcal infections occur annually, which results in healthcare costs of about $135 million [2,3]. Globally, the World Health Organization (WHO) estimates indicate that over 82 million people were newly infected with gonorrhea in 2020 [4,5]. Given that many *N. gonorrhoeae* infections are asymptomatic, reported cases likely represent only a fraction of the true prevalence [6–11].

In addition to the high incidence rate of *N. gonorrhoeae* infections, the uprising antibiotic resistance rates in *N. gonorrhoeae* have become a serious public health concern. Hence, *N. gonorrhoeae* is classified by both the WHO and the CDC as a superbug and an urgent threat [12]. *N. gonorrhoeae* has developed resistance to nearly all FDA-approved therapies, including the last resort therapeutic for *N. gonorrhoeae* infections, ceftriaxone [13–16]. Worrisomely, *N. gonorrhoeae* resistance was extended to gepotidacin which is currently in clinical trials and has not been approved yet [17,18]. These rising resistance rates underscore the urgent need for novel anti-*N. gonorrhoeae* therapeutics.

Brilacidin is a synthetic peptide with demonstrated antifungal [19,20], antiviral [21–24], and antibacterial activity, particularly against the *Staphylococcus aureus* [25–27]. It has completed phase 2 clinical trials for treating *S. aureus* skin infection (NCT02052388), SARS-CoV-2 infections (NCT04784897), and as a rinse to treat oral mucositis (NCT02324335). However, brilacidin’s activity has not been evaluated against *N. gonorrhoeae.* Given the dearth of new anti-gonococcal therapeutics and the increased interest in repurposing brilacidin for treatment of microbial infections, the aim of this study is to investigate the anti-*N. gonorrhoeae* activity of brilacidin. We assessed the anti-gonococcal activity of brilacidin against multiple multidrug-resistant *N. gonorrhoeae* strains. Additionally, we examined its killing kinetics via a time-kill assay, cytotoxicity on endocervical cells, and hemolytic activity on the human red blood cells (RBCs). Brilacidin’s ability to clear intracellular *N. gonorrhoeae* within endocervical cells was also investigated. Finally, its mechanism of action was explored using ATP leakage and propidium iodide uptake assays.

## 2. Material and methods

### 2.1. Bacterial strains and reagents

*N. gonorrhoeae* strains were obtained from the CDC, the WHO, and the American Type Culture Collection (ATCC) (Table 1). The ME-180 cell line (ATCC HTB-33) was obtained from the ATCC. Antibiotics used in this work were purchased commercially: ciprofloxacin (Sigma-Aldrich, St. Louis, MO, USA), gentamicin (Chem-Impex International, Wood Dale, IL, USA), azithromycin, and ceftriaxone (TCI America, Portland, OR, USA), and brilacidin (MedChemExpress, Monmouth Junction, NJ, USA). Media and reagents including McCoy’s 5A medium and hematin (Sigma Aldrich, St. Louis, MO, USA), triton X-100 (Acros Organics, Fair Lawn, NJ, USA), BacTiter-Glo reagent (Promega Corporation, Madison, WI, USA), Propidium iodide (PI) and nicotinamide adenine dinucleotide (NAD) (Chem-Impex International, Wood Dale, IL, USA), MTS (3-(4,5-dimethylthia- zol-2-yl)-5-(3-carboxymethoxyphenyl)-2-(4-sulfophenyl)-2H-tetrazolium) (Abcam, Waltham, MA, USA), and brucella broth, chocolate II agar plates, IsoVitaleX and bovine hemoglobin (Becton, Dickinson and Company, Cockeysville, MD, USA), were obtained from chemical vendors.

**Table 1:** MICs of brilacidin and control antibiotics against *N. gonorrhoeae* strains.

*N. gonorrhoeae* strains and description	MIC (µg/mL)				
	Brilacidin	Ciprofloxacin	Tetracycline	Azithromycin	Ceftriaxone
**CDC 166** Resistant to tetracycline, penicillin, and ciprofloxacin	8	16	4	1	0.064
**CDC 171** Resistant to tetracycline, penicillin, and ciprofloxacin	4	16	4	0.5	0.032
**CDC 172** Resistant to tetracycline, penicillin, and ciprofloxacin	4	16	2	1	0.032
**CDC 173** Resistant to tetracycline, penicillin, and ciprofloxacin	4	16	4	0.5	0.064
**CDC 174** Resistant to tetracycline, penicillin, and ciprofloxacin	4	32	4	2	0.064
**CDC 175** Resistant to azithromycin	1	≤ 0.25	1	8	0.004
**CDC 177** Resistant to tetracycline	1	≤ 0.25	2	1	0.008
**CDC 178** Resistant to tetracycline, penicillin, and ciprofloxacin	4	16	8	1	0.032
**CDC 181** Resistant to tetracycline and azithromycin	2	≤ 0.25	2	>64	0.032
**CDC 182** Resistant to tetracycline, penicillin, and ciprofloxacin	8	16	4	1	0.032
**CDC 194** Resistant to penicillin, not susceptible to ceftriaxone, cefixime and cefpodoxime	2	≤ 0.25	1	1	0.125
**CDC 202** Resistant to azithromycin	8	≤ 0.25	1	16	0.004
**WHO-F** Origin: Canada, 1991	8	≤ 0.25	0.5	0.125	≤ 0.004
**WHO-K** Origin: Japan, 2003 Resistant to tetracycline, penicillin, and ciprofloxacin	8	>64	2	0.5	0.032
**WHO-M** Origin: Philippines, 1992 Resistant to tetracycline, penicillin, and ciprofloxacin	8	2	2	0.25	0.032
**WHO-P** Origin: USA, Unknown Resistant to tetracycline and azithromycin	4	≤ 0.25	1	4	≤ 0.004
**WHO-U** Origin: Sweden, 2011 Resistant to tetracycline and azithromycin	4	≤ 0.25	1	4	≤ 0.004
**WHO-V** Origin: Sweden, 2012 Resistant to tetracycline, ciprofloxacin, penicillin, and azithromycin	4	>64	4	>64	0.125
**WHO-W** Origin: Hong Kong, 2007	2	>64	4	0.5	0.032
**WHO-X** Origin: Japan, 2009 Resistant to tetracycline, ciprofloxacin, penicillin ceftriaxone and cefixime	8	>64	4	0.5	1
**WHO-Z** Origin: Australia, 2013 Resistant to tetracycline, ciprofloxacin, penicillin, ceftriaxone and cefixime	8	>64	4	0.5	0.25
**FA1090** Isolated from patient with disseminated gonococcal infection Resistant to streptomycin	1	≤ 0.25	≤ 0.5	0.125	≤ 0.004
**MIC**_**50**_	4	16	2	1	0.032
**MIC**_**90**_	8	>64	8	>64	1

### 2.2. Antibacterial susceptibility analysis

The inhibitory activity of brilacidin and standard antibiotic drugs (ciprofloxacin, tetracycline, azithromycin, and ceftriaxone) was evaluated against 22 antibiotic-resistant *N. gonorrhoeae* strains using the broth microdilution method, as described elsewhere [28–31]. Briefly, *N. gonorrhoeae* colonies were collected and diluted in brucella supplemented broth to achieve a concentration of ~1 × 10^6^ CFU/mL. Brilacidin and control antibiotics were then serially diluted in brucella supplemented broth across 96-well plates. Plates were incubated at 37 °C with 5% CO_2_ for 24 h to determine the minimum inhibitory concentrations (MICs).

### 2.3. Time-kill kinetics

The bactericidal activity of brilacidin against *N. gonorrhoeae* FA1090 was evaluated by assessing bacterial growth kinetics, as previously described [32,33]. Briefly, a logarithmic phase bacterial culture was diluted in the supplemented brucella broth to a final concentration of ~1 × 10^6^ CFU/mL. Brilacidin and azithromycin were each added at 4 × MIC. Bacteria treated with dimethyl sulfoxide (DMSO) served as the negative control, while azithromycin served as a control antibiotic. Cultures were incubated with test agents at 37 °C for 24 h, with aliquots taken after 0, 2, 4, 6, 8, 10, 12, and 24 h, diluted and plated on chocolate II agar plates to determine the CFU.

### 2.4. Intracellular bacterial clearance assay

The intracellular bacterial clearance assay was performed to assess brilacidin’s ability to penetrate endocervical cells and eliminate the intracellular *N. gonorrhoeae*, as described elsewhere [29,34,35] with modifications. Briefly, the human endocervical epithelial cells (ME-180) were seeded into 96-well plates with McCoy’s 5A medium supplemented with 10% fetal bovine serum. ME-180 monolayers were then infected with *N. gonorrhoeae* FA1090 (multiplicity of infection (MOI) = 10) and incubated at 37°C with 5% CO_2_ for 24 h. Then, the phosphate-buffered saline (PBS) containing 320 μg/mL gentamicin was used to wash the wells three times before incubating with media containing gentamicin for one hour to kill the extracellular bacteria. Thereafter, PBS was utilized to wash the cells and they were subsequently treated with 4 × MIC of brilacidin, ceftriaxone, azithromycin, or DMSO (negative control). Plates were incubated at 37°C with 5% CO_2_ for 24 h. After incubation, the wells were washed with PBS and lysed with 2 mM EDTA and 0.5% saponin for one minute to release the intracellular bacteria for quantification.

### 2.5. Cytotoxicity and hemolysis assays

The potential toxic effect of brilacidin was evaluated using the ME-180 cell line, as described elsewhere [36–38]. Briefly, ME-180 cells were seeded and incubated with brilacidin at various concentrations (in triplicates) for 24 h. Cell viability was measured by monitoring the change of MTS color due to NADH reduction in viable cells, recorded at an absorbance of 490 nm (OD_490_).

Brilacidin’s hemolytic activity was evaluated following previously described methods [39,40]. Single-donor human RBCs (Innovative Research, MI, USA) were suspended in PBS at the concentration of 4% v/v. Brilacidin (in triplicate) was serially diluted in PBS to final concentrations of (16, 32, 64 and 128 μg/mL) and incubated with RBCs suspension at 37°C for one hour. Triton X-100 (0.1%) was used as a positive control to induce complete hemolysis, while PBS served as a negative control. After incubation, the erythrocytes were centrifuged at 800 × g for 10 min, and the absorbance of the supernatant was measured at 540 nm to assess hemolysis.

### 2.6. Permeability assays

Propidium iodide (PI) fluorescence assay was used to assess brilacidin’s ability to damage bacterial cytoplasmic membranes [41,42]. Briefly, *N. gonorrhoeae* (1 × 10^7^ CFU/mL) was incubated with brilacidin (5× and 10 × MIC), azithromycin (10 × MIC), or triton X-100 (0.1%) in the presence of 10 μM PI for 1 hour. DMSO-treated *N. gonorrhoeae* served as a negative control. After incubation, the bacterial pellet was washed with PBS, and PI uptake was measured using a plate reader (excitation at 585 nm and emission at 620 nm).

In addition, an ATP leakage assay was used to assess the membrane integrity by measuring luminescence using the Luminescent ATP Detection Assay Kit according to the manufacturer’s instructions [43].

### 2.7. Statistical analyses

Each experiment was repeated at least twice. The GraphPad Prism 9.0 (Graph Pad Software, La Jolla, CA, USA) was used to generate the graphs and statistical analysis was conducted using one-way ANOVA (analysis of variance). Results were considered statistically significant if P-values < 0.05, and data are presented as means ± standard error of the mean.

## 3. Results and discussion

### 3.1. Anti-gonococcal activity of brilacidin

The anti-gonococcal activity of brilacidin was assessed against 22 multidrug-resistant *N. gonorrhoeae* isolates, including nine WHO reference strains with diverse resistance profiles and known phenotypic and genetic markers [44]. Brilacidin showed MIC values ranging from 1 to 8 µg/mL, inhibiting 90% of the strains (MIC_90_) at 8 µg/mL and 50% of strains (MIC_50_) at 4 µg/mL (Table 1). These strains showed high resistance levels to some control antibiotics. As illustrated in Table 1, ciprofloxacin had MIC_50_ and MIC_90_ values of 16 and >64 μg/mL, respectively, while tetracycline showed MIC_50_ of 2 and MIC_90_ of 8 μg/mL. Additionally, azithromycin displayed MIC_50_ of 1 and MIC_90_ of >64 μg/mL, and ceftriaxone presented MIC_50_ and MIC_90_ values of 0.032 and 1 μg/mL, respectively. These MICs for tetracycline, ciprofloxacin, azithromycin, and ceftriaxone align with previously reported values for these strains [44,45].

### 3.2. Killing kinetics of brilacidin

**Fig 1:**
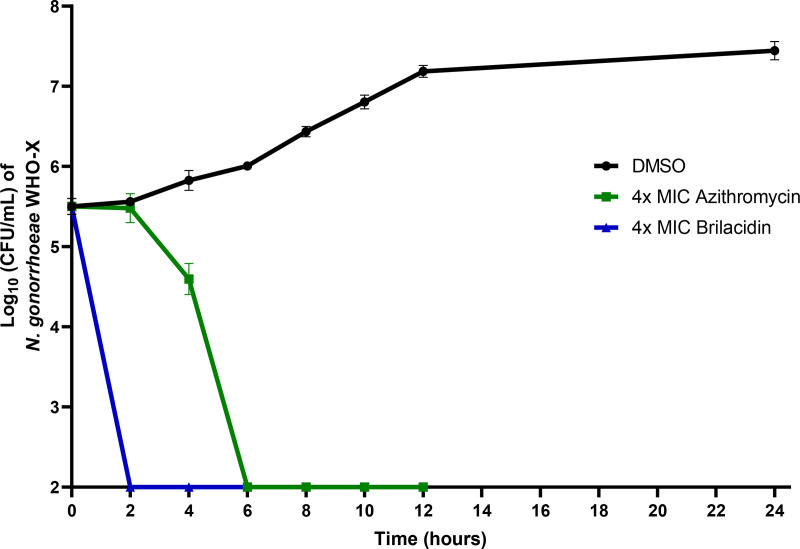
Time-kill curve illustrating the bactericidal effect of brilacidin and azithromycin (both at 4 × MIC) against N. gonorrhoeae WHO-X over a 24-hour period. Each point is the mean of Log_10_ CFU/mL, and the error bars in each point are for the standard deviation of the mean.

Brilacidin’s killing kinetics against *N. gonorrhoeae* WHO-X was evaluated in a time-kill assay. Remarkably, brilacidin (at 4 × MIC) demonstrated a rapid killing activity outperforming the control antibiotic azithromycin. As depicted in Fig 1, the burden of *N. gonorrhoeae* WHO-X was completely eradicated within 2 hours of treatment with brilacidin. However, azithromycin (at 4 × MIC) needed 6 hours to completely eradicate the *N. gonorrhoeae* burden. This rapid antibacterial activity of brilacidin is a highly desirable trait for treating *N. gonorrhoeae,* as it offers benefits such as limiting infection spread, reducing the likelihood of resistance development, shortening treatment duration and preventing disease progression which are key factors for controlling *N. gonorrhoeae* infections [46,47].

### 3.3. Cytotoxicity and intracellular clearance activity of brilacidin

The ectocervical and endocervical cells in the female reproductive tract can be infected with *N. gonorrhoeae,* allowing the bacteria to survive intracellularly. *N. gonorrhoeae* has the ability to transmigrate across mucosal epithelial cells post invasion, potentially leading to disseminated infections. It can also inhibit the autophagy process during the invasion [48–50]. Most antibiotics are ineffective at reducing the burden of intracellular bacterial infections. For instance, ceftriaxone, the drug of choice, has limited activity against intracellular bacteria due to its high molecular weight (554.58 g/mol), low active transport, and high hydrophilicity (logP = 0.6) [51]. Given these limitations, we sought to evaluate the intracellular clearance activity of brilacidin using infected human endocervical epithelial cells (ME-180).

**Fig 2:**
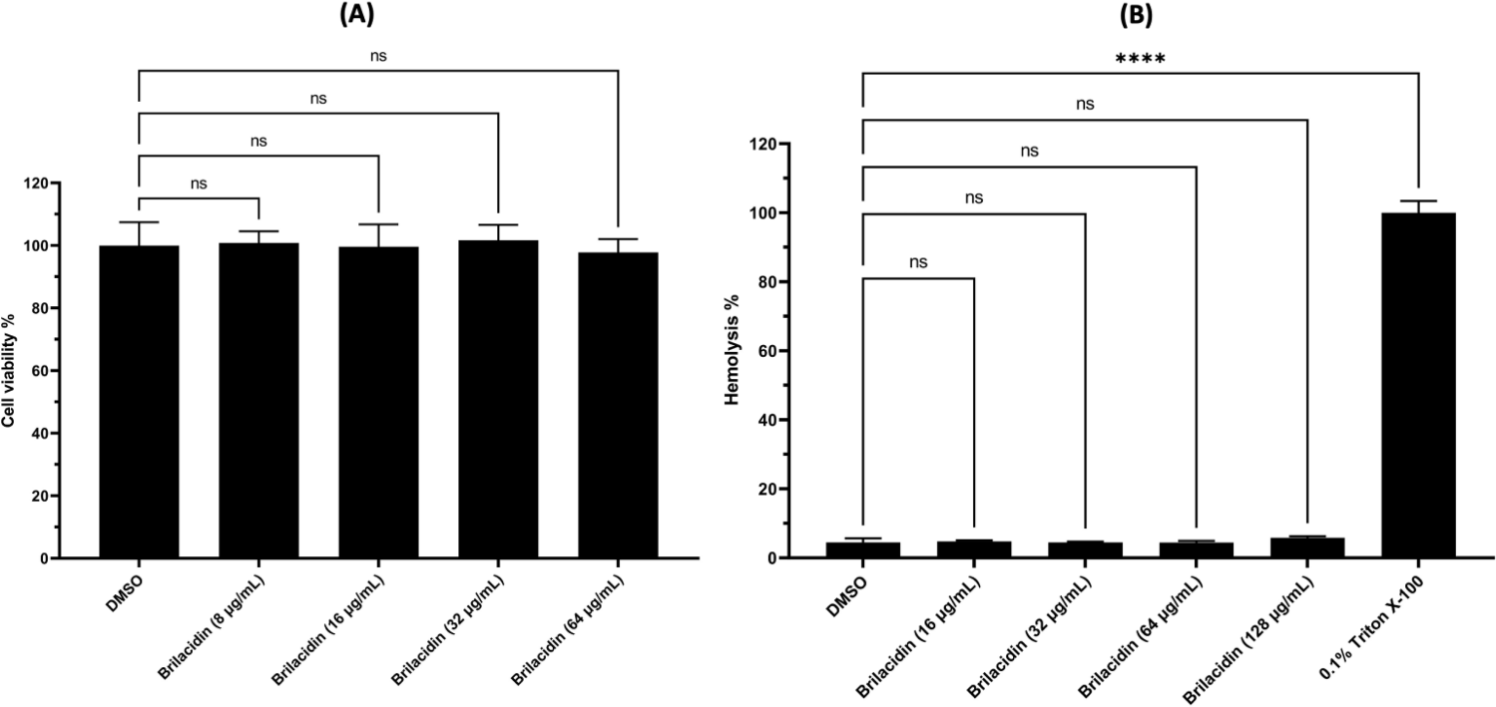
The high safety profile of brilacidin. (A) ME-180 cell viability of after incubation with different concentrations of brilacidin for 24 h. Results are shown as a percentage of cell viability relative to negative control (DMSO). (B) Hemolytic activity of brilacidin against human RBCs. The results are shown as percentage of RBCs hemolysis for each concentration of brilacidin relative to 0.1% Triton X-100 (positive control with complete hemolysis of RBCs). Error bars represent the standard deviation of the mean. **** (P < 0.0001), ns stands for not significant.

Initially, we tested the toxicity of brilacidin to endocervical epithelial cells, and found out it was well tolerated at a concentration up to 64 μg/ mL, with nearly 100% cell viability (Fig 2A). Hemolytic activity was also evaluated using human RBCs. Brilacidin showed an HC_90_ value (concentration causing 90% hemolysis) exceeding 128 μg/mL (Fig 2B), underscoring human cells’ tolerability to brilacidin. This broad therapeutic window, with minimal toxicity to mammalian cells and strong bactericidal activity, suggests brilacidin’s selectivity against *N. gonorrhoeae.*

**Fig 3:**
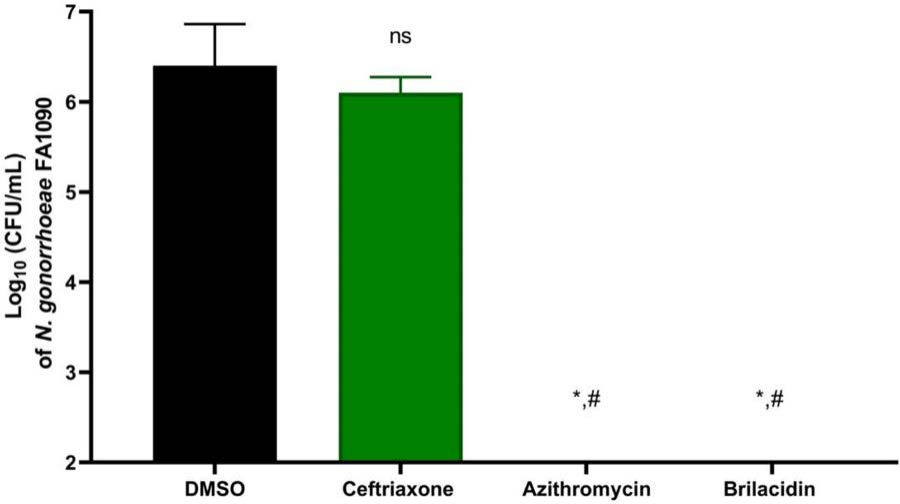
Intracellular clearance activity of ceftriaxone, azithromycin, and brilacidin (at 4 × MIC) against *N. gonorrhoeae* FA1090 in infected ME-180 cells. DMSO served as a negative control. Asterisks (*) denote statistically significant differences between test agents and DMSO (untreated) (P < 0.05). Pound signs (#) indicate statistically significant differences (P < 0.05) between brilacidin and azithromycin in comparison to ceftriaxone.

Subsequently, we evaluated brilacidin’s ability to clear the burden of intracellular *N. gonorrhoeae* within infected mammalian cells. As represented in Fig 3, brilacidin (at 4 × MIC) completely eradicated intracellular *N. gonorrhoeae* within 24 hours. Interestingly, brilacidin was superior to ceftriaxone, which showed a lower level of intracellular reduction for *N. gonorrhoeae* FA1090. These findings indicate that brilacidin can effectively penetrate host cells and eliminate *N. gonorrhoeae* at a rate superior to the drug of choice, ceftriaxone.

### 3.4. Mechanistic insights of brilacidin

**Fig 4:**
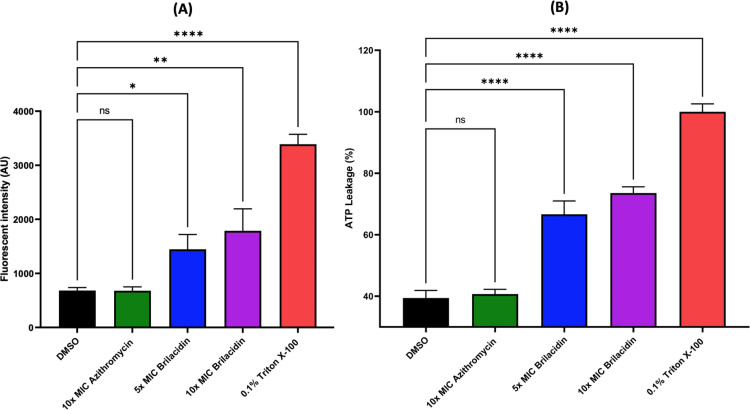
Effect of brilacidin on *N. gonorrhoeae* cytoplasmic membrane integrity and ATP leakage. A) Propidium iodide fluorescence after treating *N. gonorrhoeae* with either azithromycin or brilacidin to predict the permeabilization of the cytoplasmic membrane. B) Percentage of ATP leakage from *N. gonorrhoeae* treated with brilacidin or azithromycin relative to Triton X-100 (positive control with complete ATP leakage). Asterisks denote statistically significant differences between test agents and DMSO (untreated), * (P < 0.05), ** (P < 0.01), and **** (P < 0.0001) as determined by one-way ANOVA.

The mechanism of action of drugs with rapid bactericidal activity, particularly antimicrobial peptides, is often mediated by disrupting the bacterial membrane [52]. Since brilacidin is a peptide mimetic with rapid bactericidal activity, we sought to investigate its membrane disruption activity against *N. gonorrhoeae*. Membrane disruption was evaluated by monitoring the fluorescence intensity of propidium iodide (PI) in *N. gonorrhoeae* FA1090. As illustrated in Fig 4A, the untreated bacteria or those treated with azithromycin had no significant difference in the fluorescence intensity, indicating no disruption of the cytoplasmic membrane integrity. In contrast, brilacidin’s treatment led to a significant fluorescence increase (intensity of ~1446 and 1787 at 5× and 10 × MIC, respectively), suggesting significant membrane disruption.

Additionally, intact bacterial cells normally retain ATP, and extracellular ATP leakage indicates membrane disruption [53,54]. To verify the findings of the PI uptake assay, we measured the intracellular ATP level in *N. gonorrhoeae* cells after being treated with brilacidin compared to untreated bacteria. Triton X-100 (0.1%) was used as a positive control (considered as 100% ATP leakage). As demonstrated in Fig 4B, the supernatant of brilacidin-treated cells had a significant increase in the luminescent intensity compared to the untreated control, indicating a significant ATP leakage (~74% leakage).

These results align with previous reports demonstrating brilacidin’s membrane disruptive effect on various bacteria and fungi, including *S. aureus, Aspergillus fumigatus*, *Cryptococcus gattii* and *C. neoformans* [19,26,55].

## 4. Conclusion

This work demonstrated the anti-gonococcal activity of the peptide mimetic, brilacidin. Brilacidin displayed potent efficacy against multiple multidrug-resistant clinical isolates of *N. gonorrhoeae* with an MIC_50_ value of 4 µg/mL. In addition, brilacidin showed rapid bactericidal activity against the ceftriaxone-resistant strain WHO-X, outperforming azithromycin. It also outperformed ceftriaxone in clearing the burden of *N. gonorrhoeae* FA1090 inside infected mammalian cells. Mechanistically, brilacidin disrupted the gonococcal membrane, leading to ATP leakage and influx of propidium iodide inside the cells. These findings highlight the promising potential of brilacidin as a novel peptide mimetic to combat multidrug-resistant *N. gonorrhoeae*.

## Supporting information

S1 Data Data. (XLSX)
